# Applying two approaches to detect unmeasured confounding due to time-varying variables in a self-controlled risk interval design evaluating COVID-19 vaccine safety signals, using myocarditis as a case example

**DOI:** 10.1093/aje/kwae172

**Published:** 2024-07-03

**Authors:** Sophie H Bots, Svetlana Belitser, Rolf H H Groenwold, Carlos E Durán, Judit Riera-Arnau, Anna Schultze, Davide Messina, Elena Segundo, Ian Douglas, Juan José Carreras, Patricia Garcia-Poza, Rosa Gini, Consuelo Huerta, Mar Martín-Pérez, Ivonne Martin, Olga Paoletti, Carlo Alberto Bissacco, Elisa Correcher-Martínez, Patrick Souverein, Arantxa Urchueguía-Fornes, Felipe Villalobos, Miriam C J M Sturkenboom, Olaf H Klungel

**Affiliations:** Division of Pharmacoepidemiology and Clinical Pharmacology, Utrecht Institute for Pharmaceutical Sciences, Utrecht University, 3508 TB, Utrecht, The Netherlands; Division of Pharmacoepidemiology and Clinical Pharmacology, Utrecht Institute for Pharmaceutical Sciences, Utrecht University, 3508 TB, Utrecht, The Netherlands; Department of Clinical Epidemiology, Leiden University Medical Centre, 2333 ZA, Leiden, the Netherlands; Department of Data Science and Biostatistics, Julius Center for Health Sciences and Primary Health, University Medical Center Utrecht, 3584 CG, Utrecht, The Netherlands; Department of Data Science and Biostatistics, Julius Center for Health Sciences and Primary Health, University Medical Center Utrecht, 3584 CG, Utrecht, The Netherlands; Clinical Pharmacology Service, Vall d’Hebron Hospital Universitari, Vall d’Hebron Barcelona Hospital Campus, Universitat Autònoma de Barcelona, 08035, Barcelona, Spain; Faculty of Epidemiology and Population Health, London School of Hygiene and Tropical Medicine, WC1E 7HT, London, UK; Agenzia Regionale di Sanità, 50141, Florence, Toscana, Italy; Fundació Institut Universitari per a la recerca a l’Atenció Primària de Salut Jordi Gol i Gurina (IDIAPJGol), 08007, Barcelona, Spain; Faculty of Epidemiology and Population Health, London School of Hygiene and Tropical Medicine, WC1E 7HT, London, UK; Vaccine Research Department, Foundation for the Promotion of Health and Biomedical Research in the Valencian Region (FISABIO - Public Health), 46020, Valencia, Spain; Spanish Agency for Medicines and Medical Devices (AEMPS), 28022, Madrid, Spain; Agenzia Regionale di Sanità, 50141, Florence, Toscana, Italy; Spanish Agency for Medicines and Medical Devices (AEMPS), 28022, Madrid, Spain; Spanish Agency for Medicines and Medical Devices (AEMPS), 28022, Madrid, Spain; Department of Data Science and Biostatistics, Julius Center for Health Sciences and Primary Health, University Medical Center Utrecht, 3584 CG, Utrecht, The Netherlands; Agenzia Regionale di Sanità, 50141, Florence, Toscana, Italy; Fundació Institut Universitari per a la recerca a l’Atenció Primària de Salut Jordi Gol i Gurina (IDIAPJGol), 08007, Barcelona, Spain; Vaccine Research Department, Foundation for the Promotion of Health and Biomedical Research in the Valencian Region (FISABIO - Public Health), 46020, Valencia, Spain; Division of Pharmacoepidemiology and Clinical Pharmacology, Utrecht Institute for Pharmaceutical Sciences, Utrecht University, 3508 TB, Utrecht, The Netherlands; Vaccine Research Department, Foundation for the Promotion of Health and Biomedical Research in the Valencian Region (FISABIO - Public Health), 46020, Valencia, Spain; Fundació Institut Universitari per a la recerca a l’Atenció Primària de Salut Jordi Gol i Gurina (IDIAPJGol), 08007, Barcelona, Spain; Department of Data Science and Biostatistics, Julius Center for Health Sciences and Primary Health, University Medical Center Utrecht, 3584 CG, Utrecht, The Netherlands; Division of Pharmacoepidemiology and Clinical Pharmacology, Utrecht Institute for Pharmaceutical Sciences, Utrecht University, 3508 TB, Utrecht, The Netherlands

**Keywords:** pharmacoepidemiology, negative controls, quantitative bias analysis, COVID-19 vaccine safety, self-controlled risk interval design

## Abstract

We test the robustness of the self-controlled risk interval (SCRI) design in a setting where time between doses may introduce time-varying confounding, using both negative control outcomes (NCOs) and quantitative bias analysis (QBA). All vaccinated cases identified from 5 European databases between September 1, 2020, and end of data availability were included. Exposures were doses 1-3 of the Pfizer, Moderna, AstraZeneca, and Janssen COVID-19 vaccines; outcomes were myocarditis and, as the NCO, otitis externa. The SCRI used a 60-day control window and dose-specific 28-day risk windows, stratified by vaccine brand and adjusted for calendar time. The QBA included two scenarios: (1) baseline probability of the confounder was higher in the control window and (2) vice versa. The NCO was not associated with any of the COVID-19 vaccine types or doses except Moderna dose 1 (IRR = 1.09; 95% CI 1.01-1.09). The QBA suggested that even the strongest literature-reported confounder (COVID-19; RR for myocarditis = 18.3) could only explain away part of the observed effect, from IRR = 3 to IRR = 1.40. The SCRI seems robust to unmeasured confounding in the COVID-19 setting, although a strong unmeasured confounder could bias the observed effect upward. Replication of our findings for other safety signals would strengthen this conclusion.

**This article is part of a Special Collection on Pharmacoepidemiology**.

## Introduction

Self-controlled designs such as the self-controlled risk interval (SCRI) are often used in vaccine safety studies because they do not require an external control group.^1^ In addition, as the SCRI compares unexposed (control) and exposed (risk) time windows within individuals, it automatically adjusts for both measured and unmeasured time-fixed variables.^2^^,^^3^ However, the SCRI design is still sensitive to confounding by time-varying variables, especially when time between the control window and the risk window(s) increases. Time-varying variables can act as confounders when the probability of the variable being present differs between the pre- and postexposure window. In the setting of COVID-19 vaccines, several time-varying factors may fit these criteria and be either difficult to define and/or measure or unlikely to be included in the databases commonly used for vaccine safety studies. A main example is COVID-19 disease following SARS-CoV-2 infection, which is associated with myocardial injury.^4^ Mild cases of the disease might have gone under the radar, especially in the early stages of the pandemic when testing was not yet widespread. The probability of developing COVID-19 varies over time, with vaccination reducing the likelihood of developing COVID-19 after infection on the one hand and new virus variants increasing this on the other. Both virus transmission and vaccine uptake also depend on the vaccination strategies and lockdown rules in place at a certain point in time, and how well people adhere to these rules. In addition, the relationship between SARS-CoV-2 infection and the safety outcome of interest may vary as some virus strains are more aggressive than others.^5^^,^^6^

Several sensitivity analyses to identify unmeasured confounding exist,^7^^‑^^14^ including negative controls and quantitative bias analysis. The rationale behind negative controls comes from laboratory experiments, where it is common practice to test a hypothesis under situations that cannot logically be expected to create a particular (positive) result. This can be done by either removing the hypothesized causal agent (negative control exposure), or by evaluating an outcome that is causally unrelated to the exposure of interest (negative control outcome; NCO). In epidemiologic studies, this approach can be mimicked by searching for a variable that is similar to the exposure/outcome of interest in terms of its confounder structure except for its causal link with the true outcome/exposure, respectively.^8^^,^^10^ Under the assumption that unmeasured confounding is absent, running the main analysis with the negative control should give a null result, and deviation from this may signal unmeasured confounding for that particular relationship (and by extension the relationship of interest).

Bias analysis for unmeasured confounding could ask the question “how strong does the association between an unmeasured confounder and the exposure and outcome have to be to explain away the observed effect?” The E-value is the most accessible approach,^12^ but it may be too simplistic for situations with multiple unmeasured confounders.^15^ E-values are also commonly misinterpreted, and their utility in epidemiologic analyses has been questioned. For these reasons, a quantitative bias analysis that explores multiple scenarios regarding the incidence and strength of confounding may be more appropriate.^13^^,^^14^ Researchers can then consider whether unmeasured confounders that match these scenarios are likely to exist.

These methods are rarely applied to self-controlled designs. A recent study used NCOs to assess the robustness of several study designs within a vaccine safety framework.^16^ Across a selection of 93 NCOs, self-controlled designs appeared to have the smallest systematic error compared with cohort or case–control designs. However, the authors note that their findings may not be generalizable to COVID-19 vaccines because the way these were implemented differed from the vaccines they studied.^16^ We expand on their work by applying NCOs to the SCRI design using COVID-19 vaccines as the exposure of interest. In addition, we also apply quantitative bias analysis for unmeasured confounding. We use the myocarditis safety signal^17^ as a case example.

## Methods

This paper builds on previous work we performed on the risk of myocarditis associated with COVID-19 vaccines.^18^ We refer to this work for more detailed descriptions of the data sources.

### Negative control analysis

#### Data sources and study population

We analyzed five data sources originating from three European countries using the ConcePTION common data model.^19^ These comprise the Spanish *Base de Datos para la Investigación Farmacoepidemiológica en Atención Primaria* (BIFAP), *Sistema d’Informació per el Desenvolupament de la Investigació en Atanció Primària* (SIDIAP) and *La Fundación para el Fomento de la Investigación Sanitaria y Biomédica de la Comunitat Valenciana* (FISABIO), the Italian *Agenzia Regionale di Sanità della Toscana* (ARS), and the British Clinical Practice Research Datalink (CPRD) Aurum (Table S1).

The study population comprised all individuals registered in the databases on January 1, 2019, with at least 365 days of follow-up. Data on COVID-19 vaccination and outcome status were extracted for the study period running from September 1, 2020, to the end of data availability (Table S1). Per the SCRI design, only exposed cases were eligible for inclusion. We excluded individuals for whom vaccine brand information was missing, or who were vaccinated with a COVID-19 vaccine that was not one of the four vaccines of interest (see Exposure Measurement). We also excluded individuals whose outcome event occurred outside of the predefined control and risk windows.

#### Exposure measurement

The exposures of interest were doses 1-3 of the four COVID-19 vaccines authorized by the European Medicines Agency (EMA) early in 2021. These were Pfizer/BioNTech (Comirnaty), Moderna (Spikevax), AstraZeneca (Vaxzevria), and Janssen (COVID-19 Vaccine Janssen). Information on vaccine exposure was obtained from vaccination registers, clinical records, or general practice records, depending on the data source (Table S1).

#### Outcome measurement

The outcomes of interest were the first occurrence of myocarditis for the safety signal analysis and otitis externa for the NCO analysis (see below). Both outcomes were identified based on diagnosis code lists (Willame et al^20^  Table S2) and were either based on general practice records or hospital discharge information depending on the data source (Table S1). Individuals who experienced both myocarditis and otitis externa contributed to both.

#### Negative control outcome

The NCO analysis included all vaccinated individuals diagnosed with the NCO in either the control or one of the risk windows. NCOs must have two important features: (1) no causal association with the exposure of interest and (2) similar sources of confounding (eg, share the same risk factors) as the primary outcome.^8^^,^^10^ The second feature ensures that the NCO tests the same mechanisms of confounding that could be present for the true outcome. NCOs that lack this feature are of little value in detecting unmeasured confounding.^7^^,^^9^ To identify potential NCO, we therefore first searched for conditions with a risk factor profile similar to that of myocarditis. We subsequently checked these against published literature to see if we found any reports linking the NCO to any of the COVID-19 vaccines, following the approach described by Ryan et al.^21^ We selected otitis externa as the NCO because it shares important risk factors (infectious disease origin, skin conditions, underlying conditions that weaken the immune system) with myocarditis but has not been linked to any of the COVID-19 vaccines. The NCO analyses otherwise followed the base SCRI settings described below.

### Statistical analysis

We used the SCRI design, following the same approach we used in our earlier work.^18^ In short, for each case we defined a 60-day control window that started 90 days before the first vaccine dose and dose-specific 28-day risk windows. Cases were followed up during the 60-day control window and during each dose-specific risk window, the number of which could vary between cases. If the second dose was administered within the first dose risk window, the second dose took precedence, effectively censoring the first dose risk window. The third dose was defined as occurring at least 60 days after the second, so censoring of the second risk window due to overlap did not occur. Risk windows were also censored if a case died during that window.

We included a 30-day pre-exposure period to reduce the potential for event-dependent exposure. The analyses were stratified by vaccine brand and were adjusted for calendar time in 30-day time periods with a 10-day offset to limit potential collinearity. We performed a sensitivity analysis excluding all individuals diagnosed with COVID-19 during the study period. Analyses were performed using R (R Foundation for Statistical Computing) code adapted from the *SCCS* package^22^ to include multiple doses and vaccine brands. Analyses were performed locally according to each data source and subsequently pooled via random-effects meta-analysis using the *meta* package.^23^ We also evaluated the heterogeneity between data sources for each analysis.

### Quantitative bias analysis

We assumed that, essentially, an SCRI study compares the observed number of time intervals in which the exposure (*X*) and outcome (*Y*) are both present with the expected number of such intervals. Without adjustment for covariates, and with equal contributions of follow-up time for all subjects, it follows that the relative risk estimate (RR),


$$ \mathrm{RR}= (t-1) \times (Nyx/(n-Nyx)), $$

where *t* is the number of time intervals per participant, *Nyx* is the number of time intervals during which both *X* and *Y* are present, and *n* is the number of study participants. It follows that an important aspect of the effect estimates originates from the parameter *Nyx*. Thus, a sensitivity analysis for unmeasured confounding could focus on the distribution of an unmeasured confounder *U* in the intervals *Nyx* (risk window during which the outcome occurs) compared with its distribution in intervals without X and/or *Y* (ie, *n*-*Nyx*). In case of a binary unmeasured variable, this translates to the probability that *U* is present in intervals *Nyx* compared with the probability that it is present in time intervals without *X* and/or *Y*. If we assume that once present, *U* remains present, we can define the following parameters: Let *p*u0 be the probability of *U* in the control period in the absence of *Y* (*Y* = 0). Let *R*0 be the relative increase in the probability of *U* being present in the control period when *Y* = 1. Given that *U* was absent in the control period, let *p*u1 be the probability of *U* in the risk period when (*Y* = 0). Let *R*1 be the relative increase in the probability of *U* being present in the risk period when *Y* = 1.

As such, *R*0 and *R*1 denote the strength of the association between the unmeasured confounder *U* and the outcome *Y* given that *X* is absent (*R*0; control window) or present (*R*1; risk window). A high value for *R*0 means there is a high probability that the confounder *U* occurs in the control window if the outcome *Y* also occurs in the control window, so there is a strong association between *U* and *Y* in the absence of *X*. Values for *R*1 are interpreted in the same way except that they pertain to the risk window.

We defined two scenarios for *pu*0 and *pu*1. The first scenario reflects a situation where the probability of the confounder (for example, COVID-19 disease) was higher during the control window compared with subsequent risk windows (*pu*0 > *pu*1; with *pu*0 = 0.1 and *pu*1 = 0.05 respectively). The second scenario turned this around (*pu*0 < *pu*1; with *pu*0 = 0.05 and *pu*1 = 0.1 respectively). These values (*pu*0 and *pu*1) reflect the baseline probability, as it occurs in the absence of *Y*. This prevalence is then multiplied by *R*0 (control window) or *R*1 (risk window) to derive the actual probability of *U* in either window, which is subsequently used to simulate a binary variable *U* in the dataset. Both situations were inspired by the COVID-19 pandemic; in the early stages the prevalence of COVID-19 (confounder) was higher than after the start of the vaccination campaign (scenario 1), whereas later in the pandemic the prevalence rose again due to the emergence of more infectious strains, such as the Omicron variant (scenario 2). In each scenario, we fixed *R*0 at 1, 3, and 5 and considered a range of values for *R*1*.*^1^^‑^^10^

We simulated datasets of 300 patients with information on exposure, outcome, and an unmeasured confounder *U*. The incidence of the exposure and outcome was based on the incidences observed in the myocarditis safety evaluation study example. We also matched the length of the control and risk window to the case example. We fixed the unadjusted IRR at 3 and performed 1000 simulations for each scenario. For each simulation, we fitted an unadjusted Poisson regression model (without *U*) and a model adjusting for *U* to investigate the change to the estimated effect of the exposure *X* on outcome *Y* in the presence of confounder *U*. We extracted the model coefficients for both *X* and *U*, which were then averaged over the 1000 simulation runs.

All analyses were performed using *R*, version 4.0.3. The SCRI analysis code is openly available (https://github.com/VAC4EU/CVM), and the QBA code is provided in Appendix S1.

## Results

### Study population

In total, the five data sources contributed 29.6 million vaccinated individuals of whom 4505 were diagnosed with myocarditis and 531 204 with otitis externa. Approximately 50% of the study population were women and 20%-30% were younger than 30 years. Pfizer was most commonly administered in all data sources except CPRD, where Pfizer and Moderna were given equally often. The analyses included 427 myocarditis events and 31 373 otitis externa events (Table 1).

**Table 1:** Baseline characteristics of the study population stratified by data source.

	**ARS** **(*n* = 2 717 183)**	**CPRD** **(*n* = 8 424 436)**	**SIDIAP** **(*n* = 4 723 291)**	**BIFAP** **(*n* = 9 550 780)**	**FISABIO** **(*n* = 4 105 153)**
Women, %	52.3	51.6	51.4	52.5	51.5
Individuals younger than 30 years, %	20.1	25.7	27.6	25.1	23.9
*Brand of first COVID-19 vaccine dose, %*					
AstraZeneca Janssen Moderna Pfizer Novavax/unknown	12.1 2.7 15.6 69.5	48.2 < 1 3.1 48.7 0.1	13.0 5.5 13.4 68.1	11.6 4.5 13.3 70.7 < 0.1	12.6 5.2 12.4 69.7 0.1
*Individuals per COVID-19 vaccine dose, %*					
1 2 3	100 93.0 38.6	100 91.4 65.7	100 91.5 52.2	100 88.3 34.3	100 89.2 13.5
Individuals without COVID-19 during the study period, %	92.2	92.4	69.7	84.1	87.8
*No. of events within the study period (n, %)*					
Myocarditis	605 (< 0.1)	1413 (< 0.1)	898 (< 0.1)	734 (< 0.1)	855 (< 0.1)
Otitis externa	5620 (0.2)	225 904 (2.7)	22 234 (0.5)	194 714 (2.0)	82 732 (2.0)
*No. of events from the start of the control window* (*n*)					
Myocarditis Otitis externa	195 1385	603 66 583	404 8305	258 51 413	228 10 409
*No. of outcome events included in the analysis* (*n*)					
Myocarditis Otitis	61 454	166 14 686	77 1539	78 12 718	45 1976

Abbreviations: ARS, *Agenzia Regionale di Sanità della Toscana*; BIFAP, *Base de Datos para la Investigación Farmacoepidemiológica en Atención Primaria*; CPRD, Clinical Practice Research Datalink; FISABIO, *La Fundación para el Fomento de la Investigación Sanitaria y Biomédica de la Comunitat Valenciana*; SIDIAP, *Sistema d’Informació per el Desenvolupament de la Investigació en Atanció Primària*.

### Negative control outcomes

**Figure 1:**
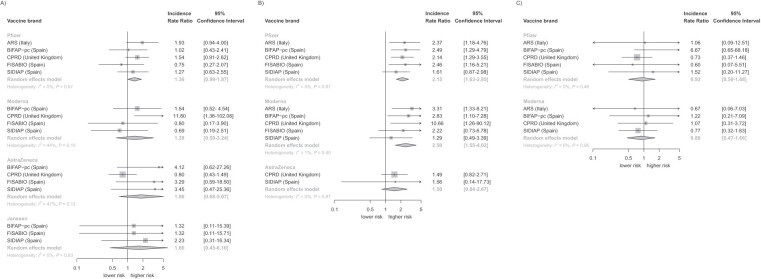
Dose-specific association between COVID-19 vaccines and myocarditis, stratified by vaccine brand and meta-analyzed across data sources. ARS, *Agenzia Regionale di Sanità della Toscana*; BIFAP, *Base de Datos para la Investigación Farmacoepidemiológica en Atención Primaria*; CPRD, Clinical Practice Research Datalink; FISABIO, *La Fundación para el Fomento de la Investigación Sanitaria y Biomédica de la Comunitat Valenciana*; SIDIAP, *Sistema d’Informació per el Desenvolupament de la Investigació en Atanció Primària*.

Myocarditis risk was elevated after both the first and second dose of each COVID-19 vaccine, but this was only statistically significant for the second dose of Pfizer (IRR = 2.15; 95% CI, 1.63-2.85) and Moderna (IRR = 2.50; 95% CI, 1.55-4.02) (Table 2). The direction of the association reversed for the third dose (IRR for Pfizer = 0.92 [95% CI, 0.50-1.68]; IRR for Moderna = 0.88 [95% CI, 0.47-1.66]), but this disappeared after excluding patients diagnosed with COVID-19 during the study period (Table 2). We observed some heterogeneity between data sources for the first doses of Moderna (*I*^2^ = 44%, *P* = 0.15) and AstraZeneca (*I*^2^ = 47%, *P* = 0.13) (Figure 1). For Moderna, this might be due to an extreme estimate from CPRD not seen in the other data sources, whereas for AstraZeneca the CPRD estimate was more conservative than the other data sources. There was little heterogeneity for the second and third dose estimates.

**Table 2:** Calendar-time adjusted risk estimates for the association between COVID-19 vaccines and selected safety (myocarditis) and negative control (otitis externa) outcomes, stratified by vaccine brand and dosing instance.

	**Myocarditis**		**Otitis externa**	
	***n*** **(risk/control)**	**IRR** **(95% CI)**	***n*** **(risk/control)**	**IRR** **(95% CI)**
*Main analysis*				
Dose 1				
Pfizer Moderna AstraZeneca Janssen	63/127 16/30 23/54 < 5/6	1.36 (0.99-1.87) 1.38 (0.59-3.24) 1.86 (0.68-5.07) 1.66 (0.45-6.16)	5234/11 710 914/1433 2421/5605 292/401	1.01 (0.92-1.10) 1.09 (1.01-1.09) 1.08 (0.90-1.28) 1.21 (0.97-1.50)
Dose 2				
Pfizer Moderna AstraZeneca	110/119 44/37 22/44	2.15 (1.63-2.85) 2.50 (1.55-4.02) 1.50 (0.84-2.67)	5788/10 460 920/1467 2430/5167	1.00 (0.96-1.03) 1.05 (0.97-1.15) 0.97 (0.88-1.06)
Dose 3				
Pfizer Moderna AstraZeneca	22/54 17/34 < 4/6	0.92 (0.50-1.68) 0.88 (0.47-1.66) –^a^	3226/6613 1283/2786 < 5/11	0.96 (0.91-1.02) 0.92 (0.91-1.05) 0.73 (0.23-2.31)
*Analysis excluding patients diagnosed with COVID-19 disease during the study period*				
Dose 1				
Pfizer Moderna AstraZeneca Janssen	48/84 14/24 19/35 < 5/< 5	1.53 (1.06-2.23) 1.48 (0.43-5.18) 1.88 (0.56-6.34) 2.16 (0.53-8.90)	4287/9774 741/1136 2154/5023 239/324	1.02 (0.91-1.14) 1.12 (1.02-.123) 1.10 (0.89-1.37) 1.18 (1.00-1.40)
Dose 2				
Pfizer Moderna AstraZeneca	91/83 36/30 18/29	2.58 (1.87-3.56) 2.72 (1.07-6.89) 1.93 (1.00-3.73)	5030/9107 788/1206 2183/4673	1.01 (0.97-1.07) 1.08 (0.99-1.19) 0.97 (0.91-1.03)
Dose 3				
Pfizer Moderna AstraZeneca	17/34 15/23	1.17 (0.46-2.98) 1.23 (0.60-2.53) –^a^	2916/5982 1139/2463 <5/10	0.95 (0.82-1.09) 0.86 (0.79-0.93) 0.80 (0.25-2.54)

Abbreviation: IRR, incidence rate ratio.

^a^ There were insufficient data (no individuals in either risk or control window) to estimate this IRR.

**Figure 2:**
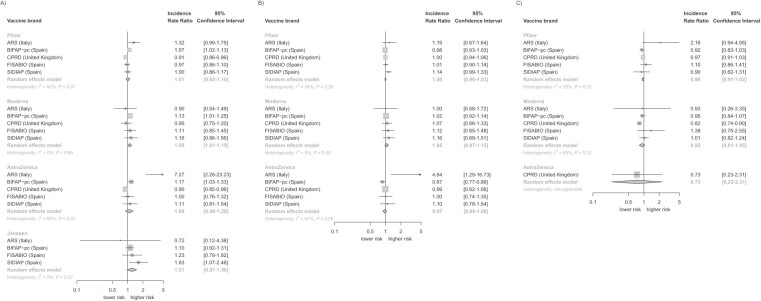
Dose-specific association between COVID-19 vaccines and otitis externa, stratified by vaccine brand and meta-analyzed across data sources. ARS, *Agenzia Regionale di Sanità della Toscana*; BIFAP, *Base de Datos para la Investigación Farmacoepidemiológica en Atención Primaria*; CPRD, Clinical Practice Research Datalink; FISABIO, *La Fundación para el Fomento de la Investigación Sanitaria y Biomédica de la Comunitat Valenciana*; SIDIAP, *Sistema d’Informació per el Desenvolupament de la Investigació en Atanció Primària*.

There was no association between the first doses of Pfizer, AstraZeneca, or Janssen and otitis externa (Table 2). For these brands, the IRR centered around the line of no effect (IRR = 1) for all vaccine brands in the first dosing instance with tight 95% CIs, except for Janssen (IRR = 1.21; 95% CI, 0.97-1.50). We observed a slight increase in risk after Moderna dose 1 (IRR = 1.09; 95% CI, 1.01-1.19). We did not observe any associations between the second or third vaccine doses and otitis externa, except for a possible protective effect for AstraZeneca dose 3 (IRR = 0.73; 95% CI, 0.23-2.31). However, this was based on only CPRD data (Figure 2). Excluding patients with at least one COVID-19 diagnosis during the study period did not significantly change our results although the protective effect for Moderna dose 3 became statistically significant (IRR = 0.86; 95% CI, 0.79-0.93). There was significant heterogeneity in the estimates for Pfizer dose 1 (*I*^2^ = 82%, *P* < 0.01), AstraZeneca dose 1 (*I*^2^ = 85%, *P* < 0.01), and AstraZeneca dose 2 (*I*^2^ = 57%, *P* = 0.05). There was moderate heterogeneity for Pfizer dose 2 (*I*^2^ = 24%, *P* = 0.26) and dose 3 (*I*^2^ = 26%, *P* = 0.25), and Moderna dose 3 (*I*^2^ = 45%, *P* = 0.12). Some of this can be explained by the ARS estimates, which were more extreme than the other databases. Removing the ARS data from the meta-analysis did not markedly change our results (data not shown).

### Quantitative bias analysis

**Figure 3:**
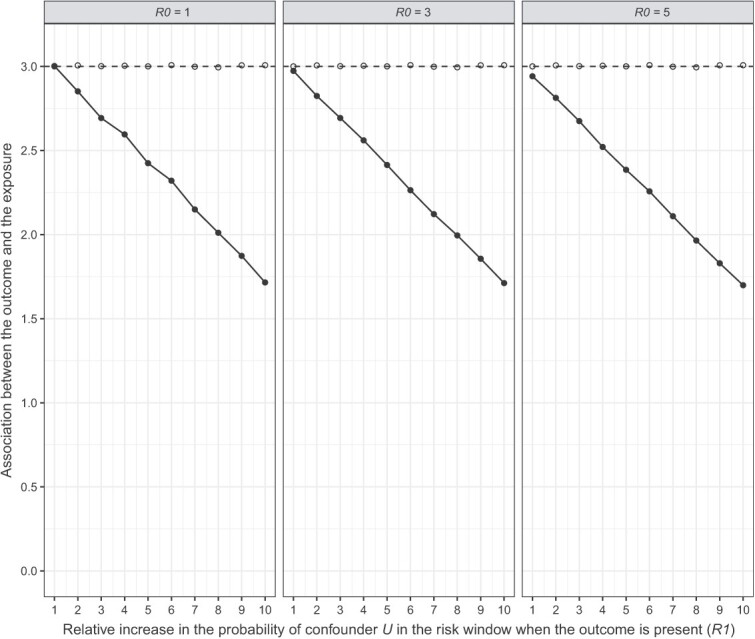
Quantitative bias analysis scenario 1: The baseline probability of confounder *U* is higher in the control window.

**Figure 4:**
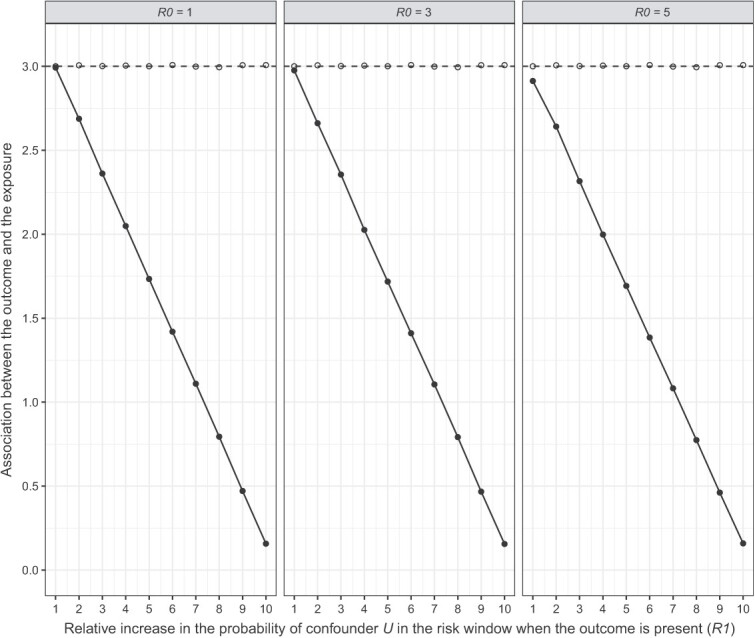
Quantitative bias analysis scenario 2: The baseline probability of confounder *U* is higher in the risk window.

For both scenarios, we observed that *R*1 biased the effect more strongly than *R*0. Given *R*1 = 1, the adjusted effect across the three levels of *R*0 went from adjusted IRR (aIRR) = 3.00 to aIRR = 2.94 in scenario 1 (Figure 3) and from aIRR = 2.99 to aIRR = 2.91 in scenario 2 (Figure 4). On the other hand, given *R*0 = 1, the adjusted effect across the levels of *R*1 went from aIRR = 3.00 to aIRR = 1.72 in scenario 1 (Figure 3) and from aIRR = 2.99 to aIRR = 0.16 in scenario 2 (Figure 4). Thus, confounder *U* could not fully explain away the fixed observed effect (IRR = 3) in scenario 1 even at the most extreme levels of *R*1 and *R*0, whereas in scenario 2 the observed effect did cross the line of no effect (IRR =1) at *R*1 > 7 regardless of *R*0. Thus, a strong association between a confounder and the outcome in the risk window could explain away at least part of the observed effect, or in other words, the presence of such an unmeasured confounder would lead to overestimation of the true effect.

**Figure 5:**
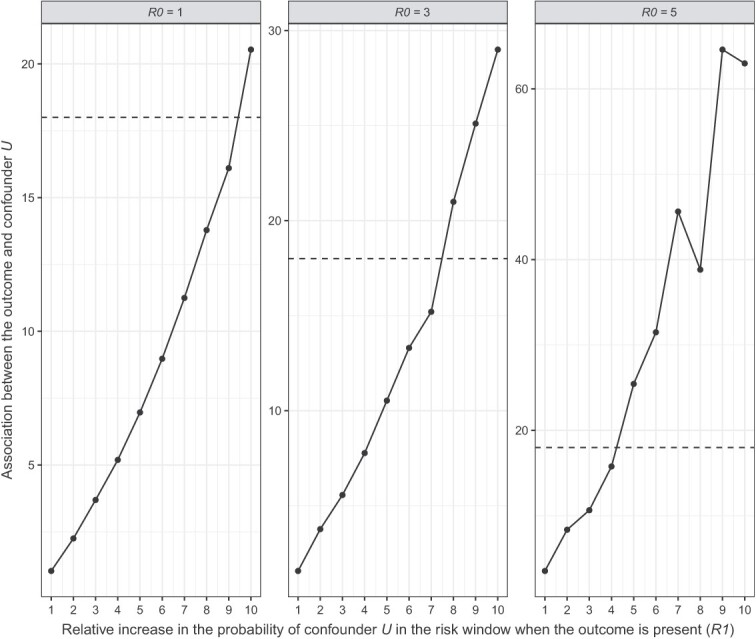
Comparing the result of quantitative bias analysis scenario 1 against the strongest confounder reported in literature (dotted line).

**Figure 6:**
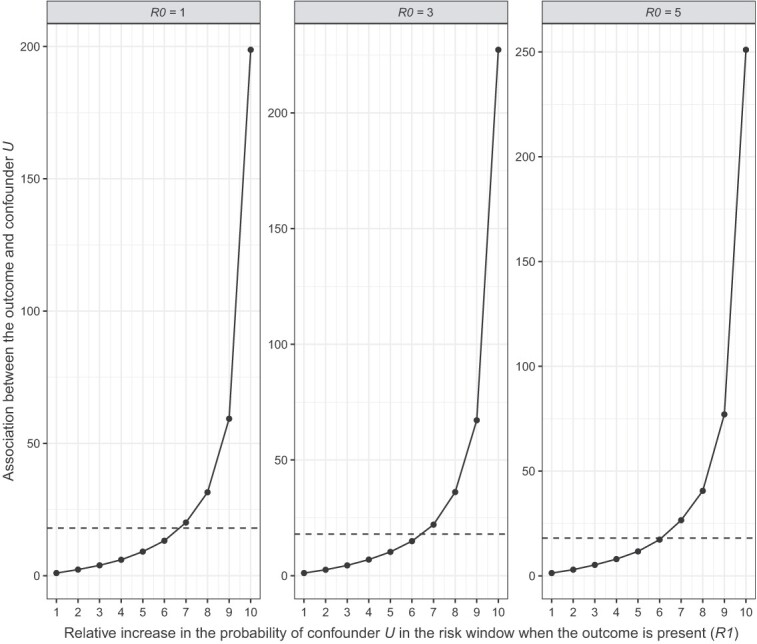
Comparing the result of quantitative bias analysis scenario 2 against the strongest confounder reported in literature (dotted line).

To properly interpret this finding, we need to consider how realistic the presence of such a confounder is. The strongest reported association between myocarditis (outcome) and COVID-19 disease (confounder), is 18.3 (95% CI, 4.0-25.1).^24^ In scenario 1, the strength of confounder *U* surpassed this value only at high *R*1 values when *R*0 = 1, but increasingly earlier as *R*0 increased (Figure 5; dotted line represents the literature-reported association). Conversely, in scenario 2 the confounder strength remained below 18.3 up until *R*1 = 6 for all levels of *R*0 (Figure 6). At this *R*1, the aIRR was approximately 1.40 for all three levels of *R*0. Thus, any realistic confounder *U* would at most explain away half of the observed effect in our situation where IRR = 3.

## Discussion

Our negative control analysis, using otitis externa as the NCO, suggests that the increased risk of myocarditis observed after the second dose of both mRNA vaccines is unlikely to be affected by unmeasured confounding. The quantitative bias analysis suggests that any realistic unmeasured confounder would at most explain away half of the association, and may thus lead to overestimation of the effect but would not lead to a false negative result. We also observed an elevated myocarditis risk after the first dose of all COVID-19 vaccines, although these were not statistically significant. The negative control analysis for the first dose returned IRRs that were all above the line of no effect, which was statistically significant for the Moderna vaccine and most extreme for the Janssen vaccine. This suggests that the first dose myocarditis estimates may have been biased slightly upward due to unmeasured confounding, especially for these two vaccine brands.

### Negative control outcomes

We show that the SCRI design is robust to unmeasured confounding within the COVID-19 vaccine setting. This is in line with earlier work^16^ showing that self-controlled designs like the SCRI are less vulnerable to bias due to (unmeasured) confounding. We expand on this work by showing that this conclusion holds true for SCRIs using one pre-exposure control window and multiple dose-specific risk windows. This addition is relevant for all SCRIs evaluating exposures where dose-specific risk windows may be too close together to allow for a separate second control window, which happened with the COVID-19 vaccine first and second doses.^18^ In addition, in situations like the COVID-19 pandemic where rapid safety evaluation is required, using a prevaccination control window is preferred because then the data can be analyzed as soon as the risk window follow-up is completed.

We did observe some heterogeneity between data sources in the otitis externa analysis, which seemed to be driven by relatively extreme estimates from ARS. This was the only data source in our study that used emergency room visits for all diagnoses instead of primary care records. If individuals suffering from otitis externa were more likely to visit the emergency room for their symptoms after having recently received their COVID-19 vaccine compared with before receiving it, this would introduce a spurious association between COVID-19 vaccines and otitis externa. Removing the ARS estimates from the meta-analysis did not change our findings, so outliers like these are unlikely to strongly affect conclusions in meta-analysis studies like ours, including multiple data sources with different data collection processes. However, studies relying on only one data source should consider how their diagnoses are collected and whether this can influence the interpretation of a negative control analysis.

Last, excluding individuals diagnosed with COVID-19 during the study period flipped the observed protective effect of the vaccine’s third dose on myocarditis into a harmful effect as also seen in other literature.^25^ We hypothesized that there may be residual confounding by COVID-19 disease at the time of the third dose, as vaccines have lowered the transmission of SARS-CoV-2 and therefore the risk of having myocarditis as a complication of COVID-19 disease. This would introduce a spurious protective effect of vaccines on myocarditis risk, which is what we saw in both the myocarditis and the otitis externa analysis. In this scenario, excluding COVID-19 disease should shift the NCO results towards the null, but our point estimate for Moderna shifted away from the null. This may be due to chance, given that the point estimate is not statistically different from the main analysis, the effect estimates for Pfizer and AstraZeneca dose 3 did not shift away, and the effect estimates for myocarditis flipped as expected. It therefore remains likely that any protective effects of a COVID-19 vaccine on myocarditis is due to confounding by COVID-19 disease.

Importantly, myocarditis risk associated with COVID-19 vaccination is sex- and age-specific,^26^ and our previous work presents the stratified results.^18^ The myocarditis results presented here should only be interpreted in the context of the NCO analysis.

### Quantitative bias analysis

We observed that an unmeasured confounder *U* associated with the outcome *Y* in the control window could explain away part of the observed association between the exposure and the outcome, but that a (single) confounder strong enough to fully explain away the effect is unlikely to exist. In our myocarditis case study, we assume time-varying COVID-19 disease status to be the main (unmeasured) confounder, given that the SCRI design is not affected by time-fixed confounding. Literature suggests COVID-19 disease increases the risk of myocarditis, with a relative risk estimate somewhere between 8.2 and 18.3.^24^^,^^27^^,^^28^ In our quantitative bias analysis, this was not strong enough to fully explain away the effect. In addition, our second scenario had a higher potential for bias and was based on the situation later during the pandemic where more infectious strains of SARS-CoV-2, such as the Omicron variant, were predominant. According to literature, these strains seemed to cause less severe disease than previous versions, such as the Delta variant,^5^^,^^6^ and are thus less likely to be strongly associated with an outcome like myocarditis.

Our findings suggest that if a strong unmeasured confounder did exist, it would lead to overestimation of the effect. This logically ties in with the SCRI design, which includes only individuals with the outcome (cases) and then tests for each individual whether that outcome is more likely to occur before exposure (control window) or after exposure (risk window).^2^ If there is a strong association between the confounder and the outcome during the risk window, disproportionally more cases will occur during the risk window compared with the control window, biasing the effect away from the null.

### Strengths and limitations

This study used five databases from across Europe, which increased power and improved reproducibility. We used two different approaches to detect unmeasured confounding to increase the robustness of our findings. The NCO analysis makes the strong assumption that time-varying confounding is identical between the true outcome and the NCO, which is unlikely to be true. Consequently, the NCO results should be interpreted while keeping this uncertainty in mind. However, as the NCO and QBA results point in the same direction, this would not change our main conclusion.

## Conclusion

To conclude, the SCRI design is relatively unaffected by time-varying unmeasured confounding with regard to the association of COVID-19 vaccination and risk of myocarditis, but replication of our findings for other safety signals would strengthen this conclusion.

## Supplementary Material

Web_Material_kwae172

## Data Availability

The SCRI analysis code is openly available (https://github.com/VAC4EU/CVM).

## Disclaimer

The research leading to these results was conducted as part of the activities of the EU PE&PV (Pharmacoepidemiology and Pharmacovigilance) Research Network, which is a public academic partnership coordinated by the Utrecht University, The Netherlands, in collaboration with the Vaccine Monitoring Collaboration for Europe network (VAC4EU). Scientific work for this project was coordinated by the University Medical Center Utrecht. The project has received support from the European Medicines Agency under the Framework service contract nr EMA/2018/23/PE. The content of this paper expresses the opinion of the authors and may not be understood or quoted as being made on behalf of or reflecting the position of the European Medicines Agency or one of its committees or working parties.
