# Supplementary material for: Applying two approaches to detect unmeasured confounding due to time-varying variables in a self-controlled risk interval design evaluating COVID-19 vaccine safety signals, using myocarditis as a case example
Source: Am J Epidemiol. 2024 Jul 3;194(1):208–19. doi: 10.1093/aje/kwae172 (PMC11735966; doi:10.1093/aje/kwae172)
Supplement: Web_Material_kwae172 [file web_material_kwae172.zip › 20240507_Supplementary files.docx]

**Title: Applying two approaches to detect unmeasured confounding due to time-varying variables in a self-controlled risk interval design evaluating COVID-19 vaccine safety signals, using myocarditis as a case example**

**Authors:** Sophie H. Bots1, Svetlana Belitser1, Rolf H. H. Groenwold2, Carlos E. Durán3, Judit Riera-Arnau3,4, Anna Schultze5, Davide Messina6, Elena Segundo7, Ian Douglas5, Juan José Carreras8, Patricia Garcia-Poza9, Rosa Gini6, Consuelo Huerta8, Mar Martín-Pérez8, Ivonne Martin3, Olga Paoletti6, Carlo Alberto Bissacco7, Elisa Correcher-Martínez8, Patrick Souverein1, Arantxa Urchuequía8, Felipe Villalobos7, Miriam CJM Sturkenboom3, Olaf H. Klungel1

**Supplementary Files**

**Table S1.** Characteristics of the participating data sources page 1

**Table S2.** Code lists for the included outcomes page 2

**Appendix S1.** R code to run quantitative bias analysis (separate R file)

**Table S1.** Characteristics of the participating data sources

|  | IT-ARS | ES-BIFAP | ES-SIDIAP | ES-FISABIO | UK-CPRD |
| --- | --- | --- | --- | --- | --- |
| *Geographical location* | Italy,  Tuscany region | Spain, multiple regions | Spain,  Catalonia region | Spain,  Valencia region | United Kingdom |
| *Population coverage* | 100% | 100% | 80% | 100% | 20% |
| *End of data availability* | December 2021 | April 2022 | June 2022 | December 2021 | March 2022 |
| *Outcome diagnoses* | Discharge diagnoses & emergency visits | General practice & specialist information reported back | General practice & discharge diagnoses | General practice, hospital, specialist information & emergency visits | General practice & specialist information reported back |
| *Diagnosis coding system* | ICD9CM | SNOMED, ICD9CM, ICD10CM | IC10CM | ICD9CM, ICD10CM | SNOMED, RCCD2 |
| *COVID-19 vaccination* | Immunisation register | COVID-19 vaccination register | Clinical records | Vaccination Registry | GP records |
| *COVID-19 diagnoses* | COVID-19 PCR-RT register | PCR-RT tests | PCR-RT tests and diagnosis codes | PCR-RT tests and Dx codes | GP records |
| *Relevant linked information sources* | In- and outpatient pharmacy dispensing, diagnostic tests and procedures, mortality register, mental health service register | Pharmacy dispensing, COVID diagnostic test, hospital discharge diagnoses | Community pharmacy invoices, specialist referrals | Diagnostic tests and procedures, specialist referrals |  |

GP: general practitioner

**Table S2.** Code lists for the included outcomes

| Coding system | codes |
| --- | --- |
| ICD10 | H60.2, H60.8, H61.0, H61.9, H62, H62.0, H62.2, H62.4 |
| ICD10CM | B00.1, B37.84, H60.2, H60.20, H60.32, H60.50, H60.51, H60.519, H60.54, H60.549, H60.8, H60.8X, H60.8X9, H61.0, H61.00, H61.009, H61.9, H62, H62.4, H62.40 |
| ICD9CM | 054.73, 112.82, 380, 380.0, 380.00, 380.01, 380.1, 380.10, 380.12, 380.14, 380.2, 380.9 |
| RCD2 | F50.., F500., F5000, F5001, F500z, F501., F5010, F5013, F5015, F5017, F501A, F501G, F501z, F502., F5024, F50z., FyuN., FyuN1, FyuN3, FyuN5, FyuN7 |
| SCTSPA | 1083251000119105, 1083291000119100, 1083341000119102, 1083361000119103, 1083381000119107, 1083731000119105, 1084121000119108, 1088381000119103, 1088421000119107, 1088471000119108, 1088491000119109, 1088511000119104, 1088791000119101, 1088861000119104, 1089021000119108, 1089181000119108, 1090991000119100, 1091031000119106, 1091081000119107, 1091101000119100, 1091121000119109, 1091401000119105, 1091471000119100, 1091631000119100, 1091791000119107, 111856000, 111898002, 155216002, 16681000, 194194008, 194197001, 194198006, 194199003, 194201001, 194202008, 194203003, 194204009, 194207002, 194214000, 194215004, 194236002, 194664001, 194666004, 194668003, 194670007, 194672004, 21543000, 232214001, 232222008, 232223003, 232224009, 232225005, 232226006, 232227002, 232228007, 232229004, 232230009, 232231008, 232236003, 232237007, 232239005, 232240007, 232241006, 232243009, 236871000119109, 267665002, 267666001, 280221000009107, 300138002, 30250000, 33934002, 34129005, 34723002, 36292003, 363174001, 39149004, 402208007, 402697000, 402698005, 402699002, 403432008, 403433003, 405821001, 427348001, 427404005, 43275000, 45431004, 45855004, 49130001, 53295002, 53316003, 54272002, 700220003, 724635009, 72898003, 76583009, 773951007, 773955003, 79972006, 86981007, 94146005, 95812002 |
| SNOMEDCT_US | 1082991000119102, 1083871000119100, 1088951000119109, 1089181000119108, 1091471000119100, 111856000, 155209003, 155210008, 155216002, 16681000, 186551009, 187021000, 194194008, 194197001, 194198006, 194199003, 194201001, 194203003, 194206006, 194214000, 194215004, 194220004, 194236002, 194664001, 194666004, 194668003, 194670007, 194672004, 21543000, 232227002, 232228007, 236871000119109, 267666001, 267751009, 267752002, 275479004, 280221000009107, 30250000, 3135009, 33934002, 34129005, 363174001, 39149004, 402697000, 427404005, 43275000, 45855004, 49130001, 53316003, 54272002, 773951007, 86981007, 94146005, 95812002 |
